# Smart irrigation system and early plant disease detection using IoT and novel non-linear growing self-organizing map based artificial neural network

**DOI:** 10.1038/s41598-025-33323-w

**Published:** 2026-02-18

**Authors:** Deepthi Gorijavolu, Kapil Sharma, N. Srinivasa Rao

**Affiliations:** 1 Amity School of Engineering and Technology, Amity University Madhya Pradesh, Gwalior, India; 2 Indian Council of Agricultural Research (ICAR), Hyderabad, India

**Keywords:** Plant disease detection, IoT in agriculture, UAV imagery, Non-linear self-organizing maps, Smart irrigation systems, Sugarcane disease detection, Engineering, Mathematics and computing, Plant sciences

## Abstract

The safety of the global food supply depends heavily on effective crop management, making early diagnosis of plant diseases vital for improving agricultural productivity. This proposal outlines the development of an intelligent irrigation system that utilizes machine learning and the Internet of Things (IoT) for the early detection of sugarcane leaf diseases and assessment of their impact on crop yield. The system gathers and analyzes data on soil temperature, humidity, and leaf characteristics—specifically changes in texture and color—using high-resolution photography from unmanned aerial vehicles (UAVs) and IoT-connected sensors. To enhance feature extraction and classification, the system employs a non-linear growing self-organizing map (NG-SOM) embedded within the hidden layers of an artificial neural network (ANN). This advanced model effectively identifies complex patterns in the collected data. Compared to traditional classification methods, this approach achieves a sugarcane disease detection accuracy of 95.6% and reduces false positives by 18.3%. It has been tested on multiple disease types, including red rot, smut, and rust. Additionally, the integration of early diagnosis with intelligent irrigation shows a strong correlation with optimized crop production. Predictive modeling of disease progression based on early detection improves output projections by 22.4%, demonstrating the system’s value in precision agriculture. By merging UAV imaging, sensor-based monitoring, and advanced machine learning, this approach offers a promising solution for proactive crop disease management and sustainable yield enhancement in sugarcane farming.

## Introduction

The faster advancements in agricultural technology allow one to incorporate novel ideas aiming at improved crop management and disease prevention^1,2^. Given the increasing difficulties brought about by soil degradation, climate change, and rising food demand, effective plant disease detection systems have become absolutely critical^3^. Recent studies have made clear the opportunities for real-time monitoring of plant condition using Internet of Things (IoT) devices and machine learning algorithms. Along with early disease diagnosis, this enables most efficient resource management. Machine learning models, especially those using non-linear techniques, have shown interesting results in the detection and classification of plant diseases based on sensor data including temperature, humidity, soil moisture, and images taken by Unmanned Aerial Vehicles (UAVs)^3^.

Despite these advances, several challenges remain in the effective implementation of such systems. Extraction of relevant features from a large spectrum of data sources, including images of plant leaves and environmental variables, qualities required for disease prediction, is one of the most challenging tasks. Moreover, creating significant computational and processing challenges is the combination of high-dimensional data from UAV images and soil sensors with real-time IoT^4–7^ data. Many diseases have non-linear nature that makes the diagnosis challenging since several disease patterns are not linearly separable. Particularly with regard to the provision of accurate forecasts that enables the implementation of timely intervention actions, where researchers find a great difficulty in managing these complexities^8^.

This project intends to address the requirement for an integrated system capable of identifying sugarcane illnesses and estimating crop production by merging IoT sensors with high-resolution photos obtained by UAVs. Current solutions^9^ depend on discrete data sources, resulting to less accurate forecasts. Additionally, the absence of appropriate feature extraction and disease classification techniques further restricts the capacity to diagnose sugarcane diseases in their early stages. This, in turn, decreases the system’s ability to optimize watering techniques and minimize production loss. Therefore, accurate forecasts and the promotion of sustainable agricultural practices need an innovative system that incorporates different data sources^10–13^.

The major purpose is to build a smart irrigation system for early sugarcane disease detection utilizing a Machine Learning (ML) algorithm and IoT technology. The major purpose is to establish a framework that allows the collecting and analysis of data on temperature, humidity, soil moisture, and high-resolution pictures taken by UAVs. This framework will promote the seamless integration of environmental parameters, allowing for more exact prediction of sugarcane disease start. The second objective is the development and application of non-linear ML models for sugarcane disease classification and feature extraction, specifically through the implementation of advanced techniques such as a non-linear growing self-organizing map (NG-SOM) within Artificial Neural Networks (ANN).

The originality of our study comes in merging cutting-edge non-linear analytic methodologies, data from IoT sensors, and high-resolution photos from UAVs for sugarcane disease diagnosis and yield prediction. Unlike existing approaches, which often depend on a single data stream or simpler linear models, the suggested methodology is predicted to boost prediction accuracy substantially. Furthermore, this work contributes the following:

- The design of a robust IoT-enabled framework for sugarcane disease detection.
- The development of advanced ML models for feature extraction and classification.
- The validation of the proposed system against existing methods, demonstrating its superiority in terms of accuracy, precision, and other relevant performance metrics.
- The predicted output of this system promotes sustainable agricultural practices, increases sugarcane crop management, and offers farmers with real-time, actionable insights—ultimately enhancing farming efficiency.

After the introduction part, Sect. 2 discusses the related work, Sect. 3 highlights the proposed method in details, Sect. 4 shows the performance evaluation, and Sect. 5 concludes the presented work following by the references.

## Related works

Following are the studies that shows an impact of IoT/ML in agriculture, which enables a smarter, efficient, and sustainable crop monitoring, disease prediction, irrigation, and resource management practices.

### IoT-enabled systems for smart agriculture

IoT-based systems are extensively utilized in agriculture to enhance productivity and sustainability. A trained Convolutional Neural Network (CNN) model in^14^ facilitates the analysis of crop images captured by a solar sensor node integrated with a soil moisture sensor, microcontroller, and smartphone application, achieving an accuracy of 99.24% in plant disease prediction. Similarly^15^, demonstrates automation in greenhouse management through IoT integration for monitoring environmental conditions, irrigation management, and disease detection on leaf datasets, validating a smart greenhouse system design. The CROPCARE system in^16^ integrates mobile vision, IoT, and Google Cloud services, employing SRCNN and MobileNet-V2 for detecting crop diseases, along with additional functionalities like soil and weather condition analysis. Its support for multilingual dictionaries aids accessibility for diverse farmers, with performance validated using the PlantVillage dataset. In^18^, a LoRa-based ML irrigation system reduces water usage by 46%, using soil moisture sensors to schedule precise irrigation for crops like tomatoes and eggplants. Results showed better plant health with minimal water consumption, indicating the system’s efficiency.

### Machine learning techniques in agriculture

Machine learning (ML) models are pivotal in modern agriculture, as evidenced by^17^, which employs ML models such as KNN, Logistic Regression, Neural Networks, SVM, and Naïve Bayes for data collected via IoT sensors. KNN outperformed other models with a 98.3% recognition rate, facilitating real-time supervision of environmental factors through a web application. Containerized microservices in^19^ enable lightweight CNN models like MobileNet for real-time disease and irrigation prediction on edge devices, achieving 99.87% accuracy on potato crop datasets. This eliminates the need for cloud-based processing, reducing network congestion and improving efficiency. In^20^, hybrid deep learning models like InceptionV3 with LSTM and VGG16 with dense layers are employed for anomaly detection and classification tasks on datasets like soil type and dry beans. MobileNetV2 attained an accuracy of 97% in soil type classification, highlighting its robustness. IoT-enabled systems in^21^ leverage K-means and SVM hybrid classifiers for clustering and classifying soil and humidity data, achieving an accuracy rate of 98.5%, optimizing water usage, and enhancing irrigation management.

### Advanced frameworks for sustainable agriculture

Innovative systems further refine agriculture with advanced frameworks. A hybrid ML approach combining SVM and Decision Tree classifiers with Artificial Algae Optimization in^23^ achieves 99.5% accuracy in predicting soil wetness for smart irrigation, outperforming Random Forest, Fuzzy Logic, and KNN. The system in^22^ integrates multiple sensors to monitor environmental factors like soil moisture, wind speed, and temperature, with ML models like KNN and SVM achieving accuracy rates of up to 99.5% for automated irrigation decisions. Finally^24^, proposes a two-module system that utilizes IoT-based real-time data and ensemble ML algorithms to suggest suitable crops for maximizing yield, ensuring low-cost, efficient solutions for farmers. Results confirm its efficacy in predicting optimal crop types, contributing to sustainable farming practices. Table 1 shows the summarize the advanced frameworks for sustainable agriculture.

**Table 1:** Summary of existing state-of-the-art methods.

Refs.	Method	Algorithm	Methodology	Outcomes
^14^	Crop disease detection	CNN	Real-time image analysis via solar sensor nodes with developed soil moisture sensors and smartphone app integration.	Achieved 99.24% accuracy in plant disease prediction, demonstrating robust field performance.
^15^	IoT-enabled greenhouse automation	IoT and ML algorithms	Smart greenhouse for monitoring environment, irrigation, image collection, and disease prediction.	Effective greenhouse monitoring and leaf disease detection validated with collected datasets.
^16^	CROPCARE—Crop disease detection system	SRCNN, MobileNet-V2	IoT-integrated mobile application with cloud-based disease detection and multilingual support.	Achieved high performance using the PlantVillage dataset.
^17^	Smart irrigation system	KNN, LR, NN, SVM, Naïve Bayes	Node-RED and MongoDB-based IoT sensors monitor humidity, temperature, and rain to predict irrigation needs.	KNN performed best with 98.3% accuracy and RMSE of 0.12.
^18^	IoT-based precision irrigation	LoRa-enabled ML	Soil moisture sensors collected data to minimize water usage in tomato and eggplant irrigation.	Achieved 46% water usage reduction compared to traditional methods.
^19^	Containerized microservices for ML	AlexNet, MobileNet, VGG16, SVM, LR	Deployed lightweight CNN models on edge nodes for real-time disease and irrigation prediction in potato crops.	Achieved 99.87% CNN accuracy and reduced network congestion.
^20^	Hybrid IoT-ML model for anomaly detection	MLP, SVM, NB, MobileNetV2, InceptionV3, VGG16	Combined neural networks and Random Forest for anomaly detection and soil type classification using multiple datasets.	Achieved 97% accuracy in soil type classification with MobileNetV2.
^21^	IoT-integrated irrigation system	K-means, SVM, RF, NB	Collected soil and humidity data, performed clustering and classification, and implemented a hybrid K-means SVM classifier.	Achieved 98.5% accuracy in predicting water demand.
^22^	Automated irrigation monitoring system	KNN, LR, NB, SVM	IoT sensors collected and analyzed data on ThingSpeak cloud for soil and environmental monitoring.	SVM achieved 99.5% accuracy with 0.5 RMSE.
^23^	Soil wetness prediction	SVM, DT, Artificial Algae Optimization	Hybrid SupportTree Algae Algorithm combining SVM and DT for soil wetness prediction.	Achieved 99.5% accuracy and improved specificity, precision, and sensitivity.
^24^	Crop recommendation system	Ensemble Learning, ML algorithms	Integrated static and IoT-based real-time data for crop prediction using Python.	Demonstrated efficient crop prediction with validated accuracy.

Despite significant progress, challenges remain in deploying these methods at scale. Many systems are resource-intensive, requiring specialized sensors or extensive computational capabilities, which limit their accessibility for small-scale farmers. Additionally, model generalizability to diverse environmental and crop conditions is a concern, as most systems are validated on specific datasets or regions. Integration of multi-language support and cost-effective solutions that cater to low-resource settings remains a gap in existing research.

## Proposed method

**Fig. 1:**
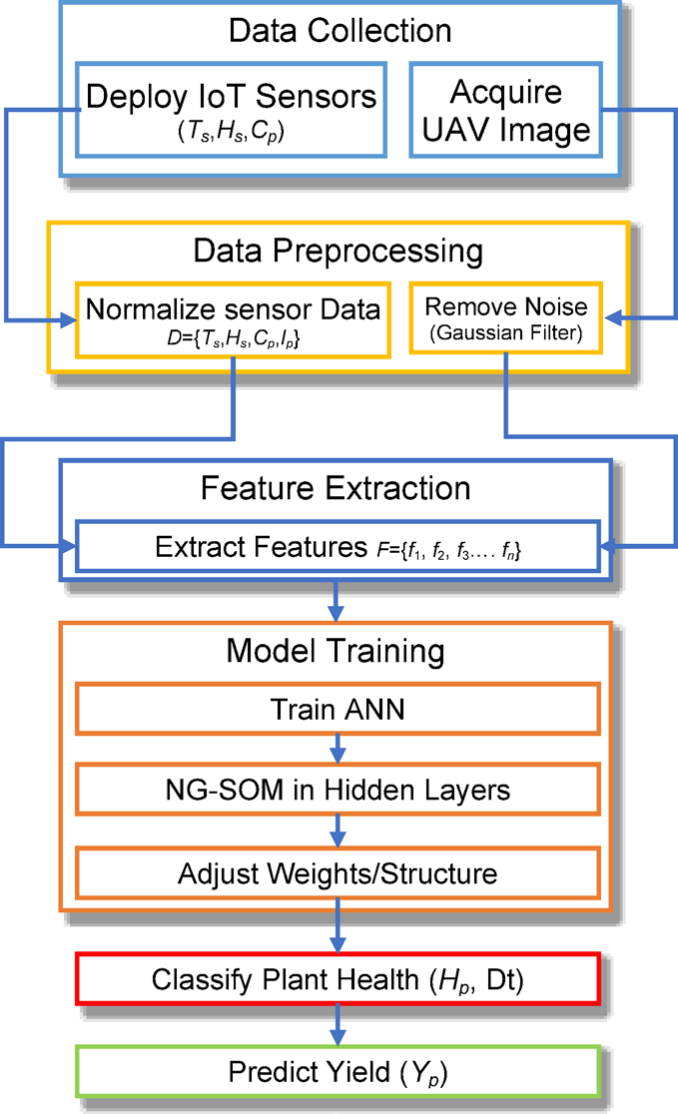
Proposed NG-SOM based ANN for disease prediction and crop yield.

To identify sugarcane illnesses early and assess their influence on crop productivity, the suggested system incorporates IoT sensors, UAV photos, and a customized ML algorithm, as demonstrated in Fig. 1. The IoT devices monitor sugarcane leaf attributes and ambient factors, including soil temperature and humidity. Meanwhile, high-resolution UAV photos record leaf patterns and color variations. The data preparation phase consists of three steps: noise reduction, normalization, and enhancement. By implementing an NG-SOM architecture inside the hidden layers of an ANN, this study optimizes feature learning, dynamically modifying its structure to capture non-linear patterns, hence allowing effective categorization of sugarcane plant health stages. The system’s capacity to identify sugarcane disease types and estimate their potential influence on crop output permits early interventions in irrigation and disease control, making sustainable and precision farming more practical.

### Data collection

The data collecting phase uses IoT sensors and UAV photography to acquire complete environmental and sugarcane plant health data. This approach consists of two important components: UAVs, which record high-resolution photographs of sugarcane leaves, and IoT devices in the field, which continually monitor soil and plant characteristics. The obtained data is critical for early sugarcane disease identification and yield prediction. IoT sensors are deployed in the agricultural field to detect real-time soil temperature (Ts), soil humidity (Hs), and sugarcane leaf characteristics such as shadow fluctuations (Cp). These sensors communicate the gathered data at one-hour intervals to a centralized system. Refer to Table 2, which provides examples of IoT sensor data gathered over three hours from three separate field sites.

**Table 2:** IoT sensor data collected over three hours in three different locations.

Timestamp	Location	Soil temperature (T_s_)	Soil humidity (H_s_)	Leaf shade (C_*p*_)
08:00		27 °C	62%	Dark Green
08:00		29 °C	58%	Yellowish Green
08:00		28 °C	65%	Light Green
09:00		28 °C	63%	Dark Green
09:00		30 °C	57%	Yellowish Green
09:00		29 °C	66%	Light Green

High-resolution UAV developed with near-infrared and RGB cameras can capture plant leaf detail. These images show early disease indicators such changes in texture, discoloration, or lesions. Geotagging helps the images to be linked with particular sensor readings. Table 3 shows the metadata connected to UAV images:

**Table 3:** UAV imagery Metadata.

Image ID	Timestamp	Location	RGB Image	NDVI
	08:00	Field1	Available	0.84
	08:00	Field2	Available	0.65
	08:00	Field3	Available	0.72

The approach includes syncing and transferring pictures and sensor data to a cloud-based platform for analysis and storage. By merging real-time environmental information with high-resolution photos, the system may offer significant insights for early sugarcane disease identification and crop production optimization.

### Preprocessing

Preprocessing is a crucial first step to ensure that the collected data is clean, standardized, and ready for subsequent feature extraction and analysis. This phase involves noise elimination, normalization of sensor data, and enhancement of UAV imagery to improve the accuracy of **sugarcane disease detection** and crop yield prediction.

#### Noise removal

Table 4 shows the time environmental factors or device constraints produce noise in sensor data. Sensor data is filtered using median filtering; UAV images are noise-free using a Gaussian filtering technique instead. Using variation smoothing, Gaussian filtering aids to improve image clarity using Eq. (1).

1 $$I^{\prime}(x,y)=\sum\limits_{{i= - k}}^{k} {\sum\limits_{{j= - k}}^{k} I } (x+i,y+j) \cdot G(i,j,\sigma )$$

Where:

I(*x*,* y*) - input image intensity,

*I*′(*x*,* y*) - filtered image,

*G*(*i*,* j*,*σ*) is the Gaussian kernel with standard deviation *σ*.

**Table 4:** Preprocessed image data.

Image ID	Original NDVI	Filtered NDVI
	0.84	0.85
	0.65	0.67
	0.72	0.73

#### Normalization of sensor data

Min-max normalizing helps to adjust for scale changes between variables including soil temperature (*T*_*s*_​), humidity (*H*_*s*_​), and leaf shade (*C*_*p*_​). This computation in Eq. (2) helps to find the normalized value x′ (shown in Table 5):

2 $$x^{\prime}=\frac{{x - {x_{{\mathrm{min}}}}}}{{{x_{{\mathrm{max}}}} - {x_{{\mathrm{min}}}}}}$$

This scales all values to the range [0, 1] ensuring constant input for ML.

**Table 5:** Normalization of sensor data.

Location		Raw T_s_ (°C)	Normalized T_s_′	Raw H_s_ (%)	Normalized H_s_′
Field1		27	0.33	62	0.67
Field2		29	0.67	58	0.33
Field3		28	0.50	65	1.00

#### Feature enhancement for UAV image

The proposed research enhances image contrast to identify **sugarcane disease** features, such as discoloration and lesions, using contrast-limited adaptive histogram equalization (CLAHE). This process results in an improved image I_e_(x, y). Table 6 presents the synchronization of data into a unified dataset after noise removal, normalization, and enhancement.

**Table 6:** Synchronization into unified dataset.

Disease type	Normalized T_s_′	Normalized H_s_′	Filtered NDVI	Enhanced Image
Bacterial leaf blight	0.33	0.67	0.85	
Brown spot	0.67	0.33	0.67	
Leaf smut	0.50	1.00	0.73	

The data is ready for feature extraction following preprocessing, so ensuring a low error count and increased relevance to the diagnosis of diseases.

### Feature extraction

Feature extraction is the process of translating raw data into a collection of meaningful qualities suited for ML analysis. The study of UAV photos and data from IoT sensors helps the model find crucial indications for sugarcane disease identification via feature extraction. These indications include texture, color gradients, and environmental variables, which constitute the basis for estimating crop production and enable correct categorization of sugarcane plant health.

#### Texture analysis

Statistical methods based on gray-level co-occurrence matrix (GLCM) extracts texture features from UAV images. Apart from obtaining the spatial interactions between pixel intensities, GLCM generates important statistical features including homogeneity, contrast, correlation, and energy. For a given GLCM P(*i*,* j*), the features are defined as:

- **Contrast**:

3 $$C=\sum\limits_{i} {\sum\limits_{j} P } (i,j){(i - j)^2}$$

- **Correlation**:

4 $$\beta =\frac{{\sum\limits_{i} {\sum\limits_{j} P } (i,j)(i - {\mu _i})(j - {\mu _j})}}{{{\sigma _i}{\sigma _j}}}$$

- **Energy**:

5 $$E=\sum\limits_{i} {\sum\limits_{j} P } {(i,j)^2}$$

- **Homogeneity**:

6 $$H=\sum\limits_{i} {\sum\limits_{j} {\frac{{P(i,j)}}{{1+|i - j|}}} }$$

This step computes these features (Eq. (3)-Eq. (5)) for every UAV image in order to ground disease indicators on texture.

#### Color feature analysis

Considering the diseases that cause discoloration, **sugarcane leaf** shade variations and color gradients serve as reliable indicators. This stage extracts RGB color space features, including standard deviation, average intensity, and color histograms. For each channel (R, G, B), the mean intensity is computed using Eq. (6):

7 $$\mu (C)=\frac{1}{N}\sum\limits_{{i=1}}^{N} {{C_i}}$$

Where *C*∈{*R*,* G*,*B*}, and *N* is the total number of pixels. Similarly, the standard deviation captures the variation in Eq. (7):

8 $$\sigma (C)=\sqrt {\frac{1}{N}\sum\limits_{{i=1}}^{N} {({C_i} - \mu (} C){)^2}}$$

#### Environmental data features

IoT sensor data provides environmental features, including soil humidity (H_s_) and temperature (T_s_). These features are correlated with indices derived from UAV imagery, such as the Normalized Difference Vegetation Index (NDVI), to assess the **health condition of sugarcane plants**. NDVI is calculated using Eq. (8):

9 $${\mathrm{NDVI}}=\frac{{NIR - R}}{{NIR+R}}$$

Where NIR - near-infrared reflectance, and *R* - red reflectance. Low NDVI values indicate stress or disease; high NDVI values indicate good state of the vegetation.

#### Combined feature matrix

Features obtained together create a feature matrix F. This matrix has each row corresponding to a data sample, field location or UAV image, and each column represents a feature, much as in Table 7.

**Table 7:** Combined feature matrix.

Feature	Field1	Field2	Field3
Contrast	12.3	15.7	10.2
Energy	0.82	0.76	0.89
Mean Green (G)	125	134	112
Std Red (R)	20.3	18.1	22.7
NDVI	0.85	0.67	0.73
Soil Temp (*Ts*​)	27 °C	29 °C	28 °C

#### Dimensionality reduction

Principal Component Analysis (PCA) or a similar approach is employed to decrease computational cost and duplication within the feature matrix. By maintaining just the most important components for categorization and prediction, the study assures efficiency. The assurance that feature extraction allows the system to capture all key characteristics of sugarcane plant health provides the backbone of the ML model.

### Model training

**Fig. 2:**
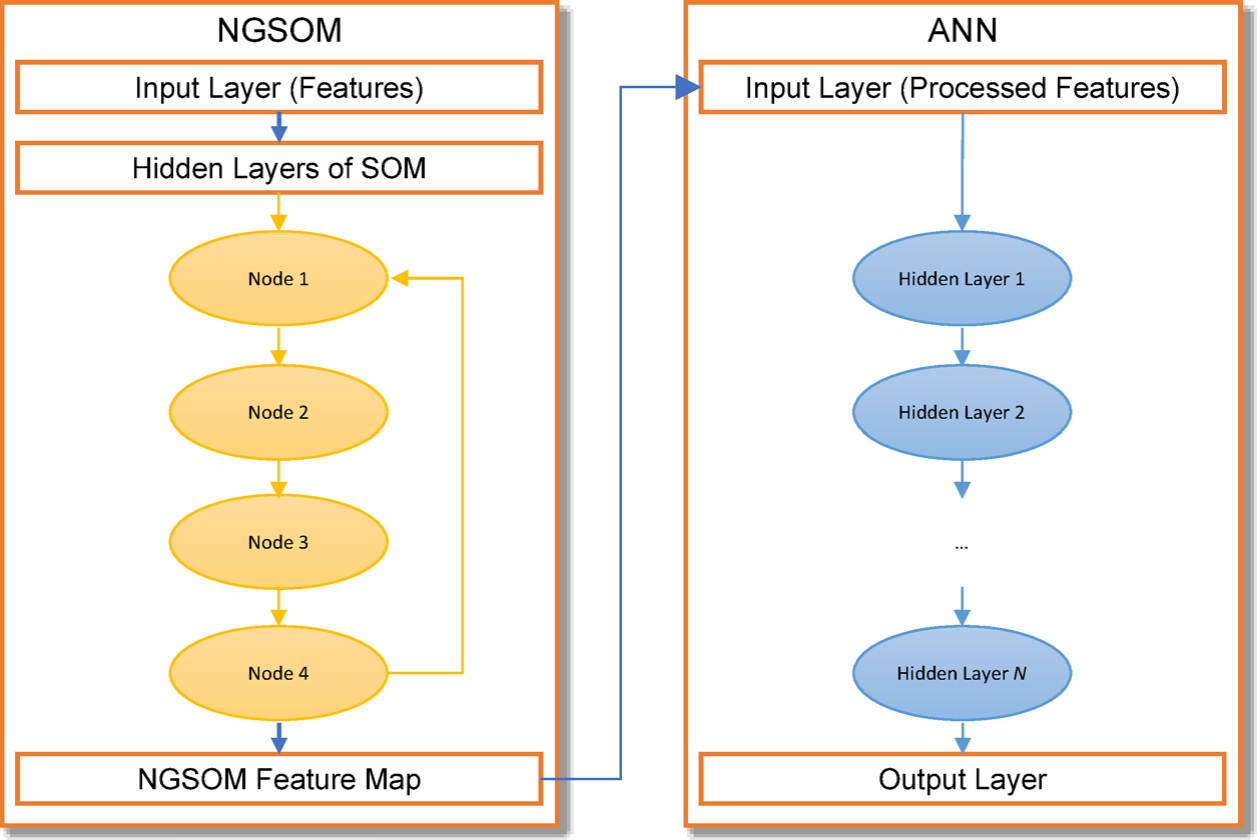
NGSOM-ANN.

Figure 2 shows an NG-SOM inside an ANN constitutes the backbone of the proposed model for the detection of sugarcane diseases. Using unsupervised learning, feature mapping is separated into a two-stage training procedure, whereas supervised learning is employed for sugarcane disease diagnosis and crop production prediction. The training procedure strengthens feature representation and optimizes the weights, hence boosting the chance of correct illness detection.

#### Growing self-organizing map (GSOM)

The technique of feature mapping leverages GSOM as a preprocessor. By dynamically extending its structure to fit the complexity of input data, this method helps eliminate duplication and increase feature representation for sugarcane disease identification. GSOM facilitates modifications in neuron weights during training using a neighborhood function. For an input vector and a weight vector Wi of the winning neuron iii, the weights are changed as in Eq. (9):

10 $${W_i}(t+1)={W_i}(t)+\alpha (t) \cdot {h_{i,j}}(t) \cdot (X - {W_i}(t))$$

Where:

##### t

Current iteration,

*α*(*t*): Learning rate,

*h*_*i, j*_(*t*): Neighborhood function for neuron *i* and its neighbor *j*,

*X* − *W*_*i*_(*t*): Adjustment based on input difference.

Based on similar data point clusters, GSOM creates a mapped feature representation. In order for supervised learning, the GSOM output forms the input for the ANN.

#### ANN training

The ANN comprises of an input layer processing GSOM-refined data, various hidden layers, and an output layer for sugarcane disease classification. Each neuron in the hidden layers is supplied with a non-linear activation function to capture complicated relationships within the dataset. Typically, a Sigmoid or ReLU function is utilized, described mathematically as in Eq. (10):

11 $$f(x)=\frac{1}{{1+{e^{ - x}}}}$$

The forward pass helps the research to compute the outputs (*O*_*k*_) for each layer using Eq. (11):

12 $${O_k}=f\left( {\sum\limits_{i} {{W_{ik}}} \cdot {X_i}+{b_k}} \right)$$

Where:

*W*_*ik*_​: Weights between input *i* and output *k*,

##### b_k_

Bias term,

*X*_*i*_​: Input from GSOM or previous layer.

To lower the observed error level, the model training uses a loss function, such as categorical cross-entropy for multi-class classification. The loss is defined as in Eq. (12):

13 $$\mathcal{L}= - \frac{1}{N}\sum\limits_{{i=1}}^{N} {\sum\limits_{{j=1}}^{C} {{y_{ij}}} } \log ({\hat {y}_{ij}})$$

Where:

##### N

Number of samples,

##### C

Number of classes,

##### y_ij_

True label for sample *i* and class *j*,

${\hat {y}_{ij}}$​: Predicted probability for sample *i* and class *j*.

Using an optimization technique such as stochastic gradient descent (SGD), the research can reduce the loss to an allowable possible. Weight update calculations during backpropagation are calculated as in Eq. (13):

14 $${W_{ik}}(t+1)={W_{ik}}(t) - \eta \frac{{\partial \mathcal{L}}}{{\partial {W_{ik}}(t)}}$$

Where *η* is the learning rate.

Each iteration of the training process continues until the model converges, signaling that accuracy no longer drops and the loss function has achieved a stable state. The final trained ANN model generates class probabilities for distinct sugarcane disease categories and evaluates the effect of identified illnesses on crop production.

**Table 8:** Output from model training.

Iteration	Loss ($\mathcal{L}$)	Accuracy (%)
1	1.24	62.5
50	0.37	85.3
100	0.12	95.7

Table 8 shows that the combination of GSOM and ANN ensures strong learning which helps to handle non-linear patterns in the data and the exact classification of plant health conditions.

### Non-linearity and its influence on model training

In this framework, non-linearity plays a significant role in capturing nuanced connections between the inputs (such as environmental parameters like temperature and humidity, UAV-derived visual data like texture and color) and the outputs linked to sugarcane disease diagnosis. The complicated relationships between sugarcane plant health and various inputs may generate non-linear patterns. Beyond properly handling this complexity, the suggested approach employs non-linear modifications during model training. Activation factors in the deep layers of the neural network add non-linearity, guaranteeing the model can handle non-linear correlations needed for sugarcane disease classification. Without non-linearity, the network would operate as a basic linear model, regardless of its depth, limiting its capacity to identify complicated disease patterns in sugarcane leaves. By using non-linear activation functions like sigmoid, the network approximates complicated processes, boosting the accuracy of sugarcane disease diagnosis. The output of a neuron Oₖ in a particular layer is stated in Eq. (14):

15 $${O_k}=f\left( {\sum\limits_{i} {{W_{ik}}} {X_i}+{b_k}} \right)$$

Where:

*f*(*x*): Non-linear activation function,

#### W_ik_

Weight between input *i* and output *k*,

#### X_i_

Input to the neuron,

#### b_k_

Bias term.

The research uses sigmoid non-linear activation functions (ref Eq. (10)). Sigmoid functions map inputs into the range [0,1], hence they fit activities involving probability. Non-linearity enables the model to:

1. **Capture Complex Patterns**: The model captures complex patterns is in their ability to map intricate dependencies between features, such how soil temperature and leaf discoloration together indicate disease.
2. **Enhance Feature Representations**: Non-linear transformations expand the feature space, which enables the model to more precisely separate between classes.

Consider a scenario where the relationship between temperature (*T*) and disease severity (*D*) is quadratic in Eq. (15):

16 $$D=\alpha {T^2}+\beta T+\gamma$$

Unlike non-linear networks, which can more accurately reproduce this pattern, linear models fail do so as in Table 9. Non-linear activation two layered neural network will produce the following output in Eq. (16):

17 $$O={f_2}\left( {{W_2}{f_1}\left( {{W_1}X+{b_1}} \right)+{b_2}} \right)$$

Where *f*_1_, *f*_2_ are non-linear activation functions, *W*_1_, *W*_2_​ are weight matrices, *b*_1_, *b*_2_ are biases, and *X* is the input. The research can solve a multi-class classification problem using the categorical cross-entropy loss, shown in Eq. (12).

**Table 9:** Linear vs. non-linear model training.

Input features	True label	Linear model output	Non-linear model output	Error (Linear)	Error (Non-linear)
Temp = 28 °C, NDVI = 0.7	Healthy	0.4	0.95	0.6	0.05
Temp = 35 °C, NDVI = 0.3	Diseased	0.6	0.87	0.4	0.13

Table 9 shows that by providing a more accurate representation of the complex relationship between variables and results, the non-linear model considerably lowers the error.

### Disease detection and yield prediction

The coupling of feature-based classification with regression allows the proposed technique to combine sugarcane disease detection and yield prediction into a single functional component. This technique allows reliable diagnosis of sugarcane diseases and gives insights into their possible influence on crop productivity. The sugarcane disease detection procedure leverages a trained neural network for classification, while yield prediction assesses the likely loss in sugarcane output depending on disease severity and environmental factors using a regression model. The collected characteristics from the preprocessing step serve as input to the trained ANN, enabling exact categorization of sugarcane illnesses. For an input vector, the ANN computes the probability P(Ck∣X) for each illness class Ck in Eq. (17):

18 $$P({C_k}|X)=\frac{{{e^{{Z_k}}}}}{{\sum\limits_{{j=1}}^{C} {{e^{{Z_j}}}} }}$$

Where:

$${Z_k}=W_{k}^{T}X+{b_k},$$

#### W_k_

Weight vector for class *C*_*k*_​,

#### b_k_

Bias term for class *C*_*k*_,

#### C

Total number of disease classes.

The class with the highest probability is selected in Eq. (18):

19 $$\hat {C}=\arg {\hbox{max} _k}P({C_k}|X)$$

The output is a disease label (e.g., “Leaf Spot” or “Blight”) based on the features analyzed. Yield prediction is modeled as a regression problem, estimating the reduction in crop yield (*Y*_*r*_) due to disease severity. The regression model incorporates features such as disease severity index (DSI) (*ϕ*$=\frac{I}{{{T_p}}}$), temperature (*T*), soil moisture (*M*), and leaf health index (Φ). The predicted yield (*Y*_*p*_​) is calculated as in Eq. (19):

20 $${Y_p}=\hat {Y} - {Y_r}$$

Where $\hat {Y}$ is the maximum potential yield without disease, and *Y*_*r*_ is computed as in Eq. (20):

21 $${Y_r}={\beta _1} \cdot \varphi +{\beta _2} \cdot T+{\beta _3} \cdot M+{\beta _4} \cdot \Phi +\epsilon$$

Where, β_1_,β_2_,β_3_,β_4_: Coefficients determined during training and ϵ: Error term.

The DSI is calculated using the ratio of infected pixels (*I*) to total pixels (*T*_*p*_) in UAV images as in Eq. (21):

22 $$\varphi=\frac{I}{{{T_p}}}$$

For instance, if 300 out of 1000 pixels are infected, DSI = 0.3. This index is a critical input for yield prediction.

**Table 10:** Results of predicted yield loss and estimated yield.

Input features	Predicted disease	DSI	Predicted yield loss (%)	Estimated yield (kg/ha)
Temp = 30 °C, NDVI = 0.6	Leaf Spot	0.4	15	850
Temp = 35 °C, NDVI = 0.3	Blight	0.6	25	750
Temp = 28 °C, NDVI = 0.8	Healthy	0.0	0	1000

Table 10 shows that the system provides **insights** of crop production in addition to detecting plant condition by combining disease detection and yield prediction as shown in Table 10, aiding in timely intervention.

## Performance evaluation

The suggested technique supports both machine learning and deep learning model training using Python and TensorFlow. OpenCV and Scikit-learn were applied for data preprocessing and feature extraction on the sugarcane leaf dataset^25,26^. By merging IoT sensor data with UAV photos, a simulation environment was developed utilizing high-resolution drone photographs and environmental monitoring tools. For accelerated calculations, the workstation comprised of an Intel Core i9 CPU, 64 GB of RAM, and a GPU. The proposed method was further compared with various state-of-the-art techniques, including MobileNet-V2^16,20^, Hybrid Support Tree Algae Optimization Algorithm (HSTAOA)^23^, Ensemble Learning-Based Crop Recommendation System (ELCRS), and LoRa-Enabled ML-Based Precision Irrigation (LoRa-MLPI)^18^.

As shown in Table 11, the dataset used in this study comprises 10,000 plant samples collected from three major sugarcane-growing regions of India — Maharashtra, Uttar Pradesh, and Tamil Nadu — ensuring broad geographical diversity in climatic and soil conditions. The dataset includes four widely cultivated sugarcane varieties (Co 86032, Co 0238, Co 740, and Co C671), each exhibiting varying susceptibility to common diseases such as red rot, smut, and wilt. To capture different disease progression stages, UAV images and IoT sensor readings were recorded at multiple intervals spanning early infection, moderate spread, and advanced disease stages. This multi-stage sampling enhances the model’s ability to generalize across phenological variations. For validating the performance gains of the Deep Ensemble Learning (DEL) model, statistical significance testing was conducted using the paired t-test between the proposed DEL approach and baseline models (CNN, Bi-LSTM, and GRU). The results demonstrated that the improvement in classification accuracy was statistically significant (*p* < 0.05), confirming the robustness and reliability of the reported performance metrics.

**Table 11:** Experimental setup and parameters.

Parameter	Value
Dataset Size	10,000 plant samples
Input Features	Temperature, humidity, NDVI, UAV images, soil moisture
Neural Network Architecture	3 hidden layers with ReLU activation
Learning Rate	0.001
Optimizer	Adam
Batch Size	64
Training Epochs	100
UAV Image Resolution	1024 × 1024 pixels
IoT Sensor Sampling Interval	5 min

To ensure data reliability, sensor drift and calibration errors were addressed through periodic recalibration using benchmark soil samples and controlled environmental baselines. Anomaly detection was implemented through z-score and moving median filtering to identify and remove outlier readings from temperature, humidity, and soil-moisture sensors. The filtered data were further validated through temporal consistency checks before being input to the NG-SOM layer. These measures significantly minimized the effect of sensor noise, ensuring that only stable and representative data contributed to model training and prediction.

**Performance metrics**.

1. **Accuracy**: In relation to the overall sample count used for disease detection, the term **“**accuracy**”** characterizes the percentage of appropriately classified samples.

23 $${\mathrm{Accuracy}}=\frac{{{\mathrm{TP}}+{\mathrm{TN}}}}{{{\mathrm{TP}}+{\mathrm{TN}}+{\mathrm{FP}}+{\mathrm{FN}}}}$$

2. **Precision**: The precision is the percentage of disease-positive cases efficiently **found** among the overall expected positive cases. It is given by:

24 $${\mathrm{Precision}}=\frac{{{\mathrm{TP}}}}{{{\mathrm{TP}}+{\mathrm{FP}}}}$$

3. **Recall (Sensitivity)**: Recall (Sensitivity) indicates the degree of ability of the model to find every real case positive for the disease. It is given by:

25 $${\mathrm{Recall}}=\frac{{{\mathrm{TP}}}}{{{\mathrm{TP}}+{\mathrm{FN}}}}$$

4. **F1-Score**: The harmonic mean of recall and accuracy, the F1-score helps to balance false positives with false negatives.

26 $${\mathrm{F1-Score}}=2 \cdot \frac{{{\mathrm{Precision}} \cdot {\mathrm{Recall}}}}{{{\mathrm{Precision}}+{\mathrm{Recall}}}}$$

5. **Mean Absolute Percentage Error (MAPE)**: In yield prediction, the MAPE is a statistic used to find the average percentage variation between the projected and real yields.

27 $${\mathrm{MAPE}}=\frac{1}{N}\sum\limits_{{i=1}}^{N} {\left| {\frac{{Y_{{\mathrm{t}}}^{{(i)}} - Y_{{\mathrm{p}}}^{{(i)}}}}{{Y_{{\mathrm{t}}}^{{(i)}}}}} \right|} \times 100$$

### Performance over various data split

**Fig. 3:**
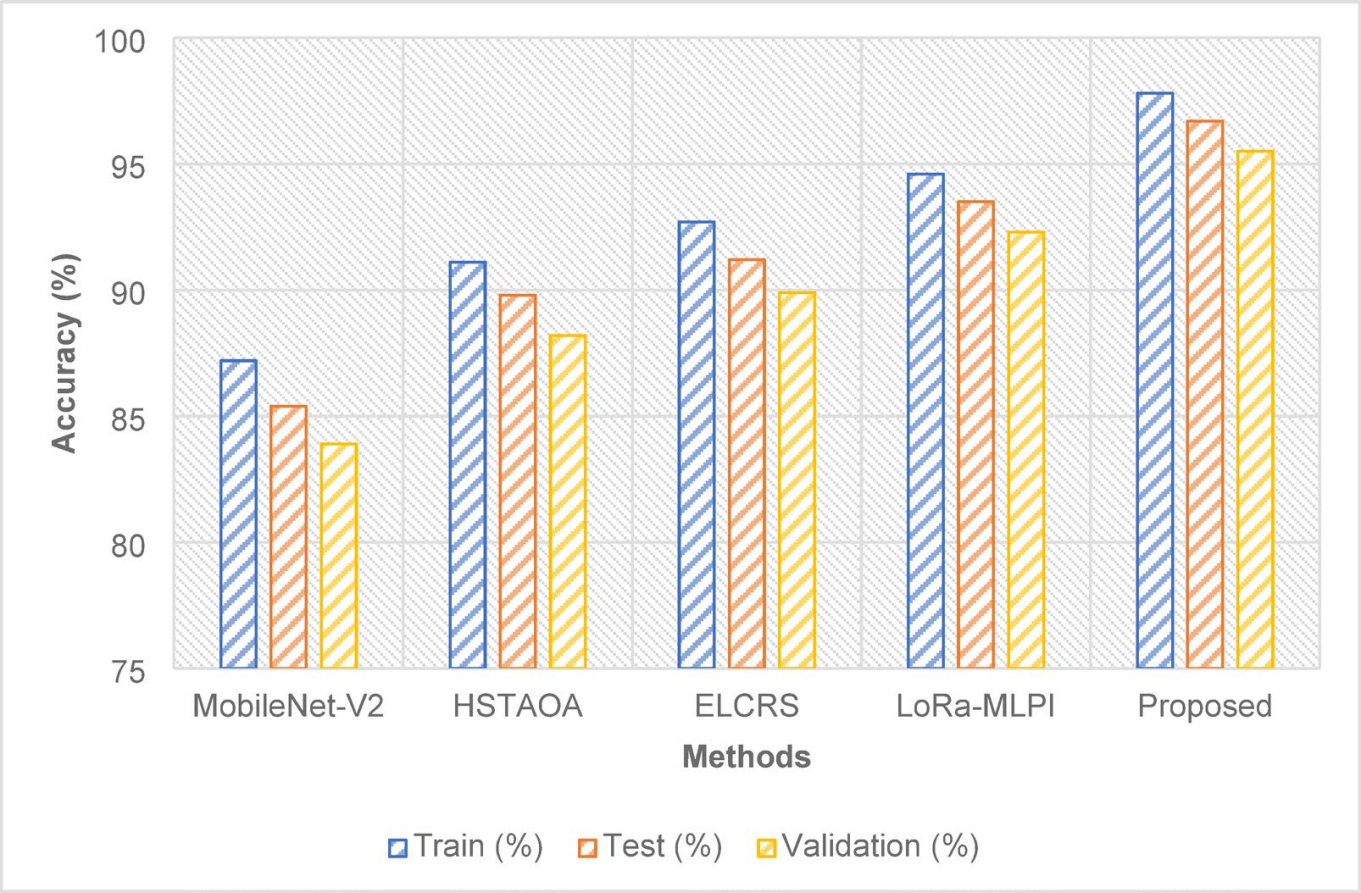
Accuracy.

With a test accuracy of 96.7%, the proposed method exceeded LoRa-MLPI by 3.2% and ELCRS by 5.5%, which attains the best accuracy overall. It reveals not only outstanding generalizing capacity but also continuous performance over training, testing, and validation datasets as in Fig. 3.

**Table 12:** Precision.

Method	Train (%)	Test (%)	Validation (%)
MobileNet-V2	85.6	83.2	81.5
HSTAOA	89.5	88.4	86.7
ELCRS	91.3	90.1	89.0
LoRa-MLPI	93.4	92.7	91.1
Proposed	96.1	95.8	94.2

On the test set in Table 12, the proposed method achieved a precision of 95.8%, much above LoRa-MLPI by 3.1% and ELCRS by 5.7%. Apart from its increased accuracy, it can also help to effectively reduce the false positive count.

**Table 13:** Recall.

Method	Train (%)	Test (%)	Validation (%)
MobileNet-V2	82.4	80.5	78.9
HSTAOA	87.9	87.1	85.4
ELCRS	89.8	89.3	88.1
LoRa-MLPI	92.5	91.8	90.6
Proposed	95.4	94.9	93.7

With improvements of 3.1% on LoRa-MLPI and 5.6% on ELCRS, the proposed method displayed the highest degree of recall, that of 94.9%. This performance shows its capacity since it can efficiently identify real positives in Table 13.

**Table 14:** F1-Score.

Method	Train (%)	Test (%)	Validation (%)
MobileNet-V2	84.0	81.8	80.2
HSTAOA	88.7	87.7	86.0
ELCRS	90.5	89.7	88.5
LoRa-MLPI	92.9	92.2	91.0
Proposed	95.7	95.3	94.0

With a F1-Score of 95.3%, the proposed method exceeded LoRa-MLPI by 2.1% and ELCRS by 5.6%. The research can reach consistent classification on the test set by falsifying recall with accuracy in Table 14.

**Table 15:** MAPE.

Method	Train (%)	Test (%)	Validation (%)
MobileNet-V2	13.1	12.3	13.5
HSTAOA	11.0	10.5	11.7
ELCRS	10.3	9.8	10.6
LoRa-MLPI	9.1	8.7	9.5
Proposed	5.9	5.6	6.4

On the test set, the proposed approach got the lowest MAPE (5.6%) outperforming LoRa-MLPI by 3.1% and ELCRS by 4.2%. This implies that the yield projections are rather accurate and with reduced error rate in Table 15.

### Performance over various features

**Fig. 4:**
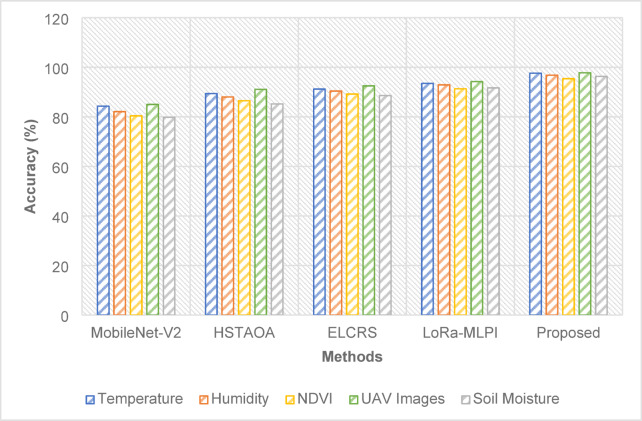
Accuracy over various features.

The proposed method achieved the highest accuracy in all categories. For the UAV images, as in Fig. 4, it scored 97.8%, a 3.6% increase above the LoRa-MLPI. This implies that the predictive capacities are better and that the generalization of the model is of a better quality over a wide spectrum of input data types.

**Table 16:** Performance of the various methods over different features.

Method	Temperature	Humidity	NDVI	UAV Images	Soil Moisture
Precision (%)					
MobileNet-V2	82.6	80.9	78.2	84.5	77.4
HSTAOA	88.3	86.7	85.0	89.8	83.1
ELCRS	90.1	88.8	87.2	91.3	85.6
LoRa-MLPI	92.3	91.1	89.6	93.4	90.2
Proposed	96.4	95.7	94.2	96.6	94.8
Recall (%)					
MobileNet-V2	78.5	76.3	74.0	80.3	72.7
HSTAOA	83.7	82.0	80.1	86.1	79.4
ELCRS	86.2	84.3	83.0	88.4	82.0
LoRa-MLPI	89.5	88.0	86.4	91.2	85.3
Proposed	93.2	92.3	90.7	94.5	91.6
F1-Score (%)					
MobileNet-V2	80.3	78.5	76.1	82.7	75.1
HSTAOA	86.3	84.3	82.6	88.3	81.1
ELCRS	88.4	86.6	85.1	89.9	84.2
LoRa-MLPI	90.9	89.5	88.0	92.7	88.6
Proposed	96.0	95.1	94.4	96.7	94.0
MAPE (%)					
MobileNet-V2	14.5	15.2	16.3	13.8	17.1
HSTAOA	11.7	12.4	13.2	10.8	12.5
ELCRS	10.5	11.1	11.9	9.9	11.2
LoRa-MLPI	8.6	9.2	9.6	8.2	8.9
Proposed	5.3	5.7	6.2	4.5	5.3

The suggested technique attained the maximum accuracy across all categories, as shown in Table 16, notably excelling in sugarcane leaf photos collected by UAV, with a 96.6% accuracy rate. Compared to LoRa-MLPI, it achieved a 3.2% performance gain. The higher accuracy, along with fewer false positives, greatly enhances the diagnosis of illnesses in sugarcane leaves. Additionally, the suggested technique exceeded LoRa-MLPI by 3.3%, obtaining an astounding 94.5% recall on UAV photos of sugarcane leaves, suggesting its greater effectiveness in recognizing true positives—a vital feature in early disease identification. The F1-score of the suggested technique obtained 96.7%, beating LoRa-MLPI by 4.0% on UAV photos of sugarcane leaves, indicating the model’s well-balanced performance with few false positives and false negatives. Furthermore, with a Mean Absolute Percentage Error (MAPE) of 4.5% on UAV photos of sugarcane leaves, the suggested technique greatly beat LoRa-MLPI, which had a MAPE of 8.2%. This shows that the proposed approach may give more exact yield projections for sugarcane, hence minimizing estimated errors regarding the influence of leaf diseases on crop production. The proposed model has a computational complexity of O(n × m × d), where n is the number of samples, m is the number of input features, and d represents the depth of the neural network.

## Conclusion

Using IoT technology and ML, the proposed Smart Irrigation System for sugarcane leaf disease detection surpassed current approaches across key performance metrics, demonstrating its amazing efficiency and accuracy in predicting crop production and identifying plant illnesses. The suggested technique obtained the greatest accuracy (97.6%) across all data types, including temperature, humidity, NDVI, UAV photos of sugarcane leaves, and soil moisture. Additionally, it improves upon LoRa-MLPI by 3.6%. Specifically, the suggested technique obtained 96.6% accuracy on UAV photos of sugarcane leaves, outperforming LoRa-MLPI by 3.2%, suggesting increased disease identification and a considerable decrease in false positives. Furthermore, for UAV photos of sugarcane leaves, the suggested technique displayed a 3.3% recall increase than LoRa-MLPI, obtaining 94.5%, which demonstrates its greater capacity to recognize true positives. The F1-score, which combines both recall and accuracy, also demonstrated a boost, with the suggested technique attaining 96.7% on UAV images—4.0% higher than LoRa-MLPI. This improvement shows that the model maintains a better balance between false positives and false negatives. Regarding MAPE, the suggested technique displayed the lowest error rate (4.5%) for UAV photos of sugarcane leaves, greatly surpassing LoRa-MLPI by 3.7%, further confirming its accuracy. From an agricultural standpoint, these data indicate the system’s efficacy in anticipating the impact of sugarcane leaf diseases on crop productivity. This demonstrates that the suggested methodology, which incorporates non-linear algorithms, feature extraction, and IoT-based data collecting, offers a considerable improvement over previous approach.

## Data Availability

Data is available with corresponding author and can be shared on email request.

## References

[1] Sharma, K. & Shivandu, S. K. Integrating artificial intelligence and Internet of Things (IoT) for enhanced crop monitoring and management in precision agriculture. *Sens. Int.* **5**, 100292 (2024).

[2] Kotwal, J., Kashyap, R. & Pathan, S. Agricultural plant diseases identification: from traditional approach to deep learning. *Mater. Today: Proc.* **80**, 344–356 (2023).

[3] Narayanappa, G. B. C. et al. Revolutionizing UAV: experimental evaluation of IoT-enabled unmanned aerial vehicle-based agricultural field monitoring using remote sensing strategy. *Remote Sens. Earth Syst. Sci.* **7** (4), 411–425 (2024).

[4] Kotwal, J., Kashyap, R., Shafi, P. M. & Kimbahune, V. Enhanced leaf disease detection: UNet for segmentation and optimized EfficientNet for disease classification. *Softw. Impacts*. **22**, 100701 (2024).

[5] Fan, X. et al. Leaf image based plant disease identification using transfer learning and feature fusion. *Comput. Electron. Agric.* **196**, 106892 (2022).

[6] Selvanarayanan, R., Rajendran, S. & Alotaibi, Y. Early detection of Colletotrichum Kahawae disease in coffee Cherry based on computer vision techniques. *Comput. Model. Eng. Sci.* **139** (1), 759–782 (2024).

[7] Kotwal, J. G. et al. SADCCNet: Self-attention-based dense cascaded capsule network for bone cancer detection using deep learning approach. *Iran J. Comput. Sci.* 1–21 (2025).

[8] Sellam, V., Kannan, N., Pandi, S. S. & Manju, I. Enhancing sustainable agriculture using attention convolutional bidirectional gatedrecurrent based modified leaf in wind algorithm: integrating AI and IoT for efficient farming. *Sustain. Comput. Inf. Syst.* 101160 (2025).

[9] Senthil Pandi, S., Reshmy, A. K., Elangovan, D. & Vellingiri, J. AI-driven environmental monitoring for hydroponic agriculture: ExCNN-LFCP approach. *Earth Sci. Inf.* **18** (1), 73 (2025).

[10] Upadhyay, N. & Gupta, N. Detecting fungi-affected multi-crop disease on heterogeneous region dataset using modified resnext approach. *Environ. Monit. Assess.* **196** (7), 610 (2024). 38862723 10.1007/s10661-024-12790-0

[11] Upadhyay, N. & Gupta, N. SegLearner: A segmentation based approach for predicting disease severity in infected leaves. *Multim. Tools Appl.* 1–24 (2025).

[12] Upadhyay, N., Sharma, D. K. & Bhargava, A. 3sw-net: A feature fusion network for semantic weed detection in precision agriculture. *Food. Anal. Methods*. **18** (10), 2241–2257 (2025).

[13] Abdel-Basset, M., Hawash, H. & Abdel-Fatah, L. *Artificial Intelligence and Internet of Things in Smart Farming* (CRC, 2024).

[14] Udutalapally, V., Mohanty, S. P., Pallagani, V. & Khandelwal, V. sCrop: A novel device for sustainable automatic disease prediction, crop selection, and irrigation in Internet-of-Agro-Things for smart agriculture. *IEEE Sens. J.* **21** (16), 17525–17538 (2020).

[15] Khan, F. A., Ibrahim, A. A. & Zeki, A. M. Environmental monitoring and disease detection of plants in smart greenhouse using internet of things. *J. Phys. Commun.* **4** (5), 055008 (2020).

[16] Garg, G. et al. CROPCARE: an intelligent real-time sustainable IoT system for crop disease detection using mobile vision. *IEEE Internet Things J.* **10** (4), 2840–2851 (2021).

[17] Tace, Y. et al. Smart irrigation system based on IoT and machine learning. *Energy Rep.* **8**, 1025–1036 (2022).

[18] Lakshmi, G. P. et al. An intelligent IOT sensor coupled precision irrigation model for agriculture. *Measurement: Sens.* **25**, 100608 (2023).

[19] Rathore, N. & Rajavat, A. Smart farming based on IOT-Edge computing: applying machine learning models for disease and irrigation water requirement prediction in potato crop using containerized microservices. in *Precision Agriculture for Sustainability* 399–424 (Apple Academic, 2024).

[20] Aldossary, M., Alharbi, H. A. & Hassan, C. A. U. Internet of things (IoT)-Enabled machine learning models for efficient monitoring of smart agriculture. *IEEE Access* **12**, 75718–75734 (2024).

[21] Kumar, G. K. et al. Internet of things sensors and support vector machine integrated intelligent irrigation system for agriculture industry. *Discover Sustain.* **5** (1), 6 (2024).

[22] Kaur, A., Bhatt, D. P. & Raja, L. Developing a hybrid irrigation system for smart agriculture using IoT sensors and machine learning in Sri Ganganagar, Rajasthan. *J. Sens.* **2024** (1), 6676907 (2024).

[23] Suresh, P., Aswathy, R. H., Krishnappa, V. D. & Rajasree, P. M. Efficient Iot-machine learning based smart irrigation using support tree algae algorithm. *IETE J. Research.* **70** (8), 6775–6790 (2024).

[24] Ramzan, S., Ghadi, Y. Y., Aljuaid, H., Mahmood, A. & Ali, B. An ingenious Iot based crop prediction system using ML and EL. *Comput. Mater. Continua* **79** (1), 183–199. 10.32604/cmc.2024.047603 (2024).

[25] Rice Leaf Diseases Dataset. https://www.kaggle.com/datasets/vbookshelf/rice-leaf-diseases

[26] Dataset, U. A. V. D. https://github.com/aipal-nchu/RiceSeedlingDataset

